# Factors Related to Recurrence Rates of Extramammary Paget Disease: A Case Series and Literature Review

**DOI:** 10.1055/a-2706-1145

**Published:** 2026-01-17

**Authors:** Boonyaporn Kotistienkul, Nattanit Poungjantaradej, Natthapong Kongkunnavat, Warangkana Tonaree

**Affiliations:** 1 Division of Plastic Surgery, Department of Surgery, Faculty of Medicine Siriraj Hospital, Mahidol University, Bangkok, Thailand

**Keywords:** extramammary Paget disease, surgical margins, pathological margins, local recurrence rates, tumor size

## Abstract

Extramammary Paget disease (EMPD) is a rare cutaneous malignant lesion. Surgical resection is the primary treatment. However, the ideal surgical margin remains uncertain. This study aimed to investigate the correlation between different margins and EMPD recurrence rates. A total of 35 EMPD patients underwent surgical resection between 2008 and 2018. This study considered multiple factors associated with the local recurrence rate, including surgical margin, depth of excision, pathological margin, tumor size, and the time until recurrence occurred. The study demonstrated that a surgical margin of 2 cm signified the outcome of local recurrence, with 20.8% in the <2 cm group and no local recurrence (0.0%) in the ≥2 cm group ( *p* = 0.157). The tumor size statistics significantly affected the local recurrence at 9 cm ( *p* < 0.05). Other managements had no statistical significance to the local recurrence rate. A surgical margin size of more than 2 cm is suggested for the wide excision of EMPD patients, considering the recurrence percentage and outcome in this study.

This retrospective study aimed to find the relation of surgical margin and the disease outcome of extramammary Paget disease (EMPD) by considering multiple factors associated with the local recurrence rate, including surgical margin, depth of excision, pathological margin, tumor size, and the time until recurrence occurred. The result showed that a surgical margin of ≥2 cm gave a better outcome of disease, and a tumor larger than 9 cm significantly affected the recurrence rate. The study suggests surgical excision of more than 2 cm around the tumor.

## Introduction

Extramammary Paget disease (EMPD) is a rare and enigmatic cutaneous malignancy characterized by the presence of neoplastic cells within the intraepidermal layers. EMPD typically manifests in areas rich in apocrine glands, such as the anogenital region, perineum, and axilla 1 2 and predominantly affects older individuals, with a higher incidence in women. 3 EMPD accounts for 7 to 14% of Paget's disease and is mostly non-invasive. 4 The origin of this disease can be divided into primary and secondary EMPD, with the secondary EMPD associated with colon cancer, urinary tract cancer, and ovarian cancer in around 5 to 42% of cases. 5 6

The management of EMPD primarily involves surgical excision, aiming for complete removal of the affected area. 7 However, determining the adequate surgical margin to achieve clear resection while preserving functionality remains a clinical dilemma. 8 Study of Mohs micrographic surgery offers precise microscopic precision of skin cancer management, including EMPD, 9 10 11 where it showed better recurrence rate in EMPD. 12

Given its complex clinical presentation, EMPD necessitates a multidisciplinary approach involving dermatologists, oncologists, pathologists, and surgeons to ensure accurate diagnosis, appropriate treatment planning, surgical margins, additional oncological treatment, and long-term follow-up care. 13 Various factors may contribute to the recurrence rate of EMPD cases. EMPD poses clinical diagnostic challenges, which often lead to delayed diagnosis and subsequent challenges in managing the disease effectively. This study provided insight into the association of the factors with the recurrence rates, including tumor size and surgical margin, compared to other prior studies.

## Case

This study recruited Thai patients with EMPD who underwent surgical resection from 2008 to 2018. We informed and obtained all patients' written consent for their demographic data and pictures, as considered by the principles outlined in the Declaration of Helsinki. The EMPD details included the type of tumor, characteristics, symptoms, location, and tumor size. The operations consisted of the surgical margin and the depth of excision. Postsurgical treatments such as chemotherapy, radiotherapy, duration until local recurrence occurs, and late follow-up, were collected.

A total of 43 EMPD cases treated with surgical resection were included, with 5 cases excluded due to insufficient data, and another 3 cases were secondary EMPD. The mean age of the cohort was 69.83 years, with a slightly predominant female population (57.1%; Table 1 ).

**Table 1:** Patient demographic data, including age, sex, body mass index, and underlying diseases

Demographic data	All (35)
Age	69.83 ± 8.51
Sex (female percentage)	20 (57.1%)
BMI	24.12 ± 4.62
DM	6 (17.1%)
HTN	21 (60.0%)
DLP	16 (45.7%)
CKD	1 (2.9%)
Other	10 (28.6%)
Previous history of cancer	9 (25.7%)

Abbreviation: BMI, body mass index; DM, diabetes mellitus; HTN, hypertension; DLP, dyslipidemia; CKD, chronic kidney disease.

The tumor data showed that the most common locations were the genital area (85.7%), perianal (2.9%), and lower extremity (2.9%). The highest presentation was plaque (57.1%), the most common border was well-defined (77.1%) and presented with ulceration for 34%. The median size of the tumors was 7.30 cm, and the median area of the tumor was 35.13 cm ^2^ ( Table 2 ). The details of operative treatment showed that wide excision was the most common surgical treatment for EMPD, and primary closure was the most common reconstruction. The surgical margins varied from 0.5, 1.0, 2.0, to 3.0 cm. Other oncological treatments included chemotherapy and radiotherapy ( Table 3 ).

**Table 2:** Tumor characteristic data, including type of tumor, stage, presentation, ulceration, border type, location, size, and area of tumor

Tumor data	All (35)
Stage of tumor	
1a	6 (17.1%)
1b	9 (25.7%)
2	4 (11.4%)
3	3 (8.6%)
Presentation	
Mass/nodule	4 (11.4%)
Patch	6 (17.1%)
Plaque	20 (57.1%)
Other	5 (14.3%)
Ulceration	12 (34.3%)
Border	
Well-defined	27 (77.1%)
Ill-defined	8 (22.9%)
Location	
Genital	30 (85.7%)
Perianal	1 (2.9%)
Lower extremity	1 (2.9%)
Combined	3 (8.6%)
Size (cm), (median [P25, P75])	7.30 [4.50, 9.50]
Area (cm ^2^ ), (median [P25, P75])	35.13 [15.30, 81.75]

**Table 3:** Operative treatment including type of operation, surgical margin, lymph node dissection, type of reconstruction, other oncological treatment, and mean follow-up time

Operative data	All (35)
Operation	
Wide excision	32 (84.2%)
Amputation	1 (2.6%)
Excision	1 (2.6%)
En bloc resection	4 (10.5%)
Surgical margin (cm)	
0.5	5 (14.3%)
1.0	19 (54.3%)
2.0	8 (22.9%)
3.0	3 (8.6%)
LN dissection	6 (17.1%)
Reconstruction	
Primary	18 (51.4%)
Graft	13 (37.1%)
Flap	4 (11.4%)
Other treatment	
Preoperative radiotherapy	1 (2.9%)
Postoperative radiotherapy	5 (14.3%)
Postoperative chemotherapy	1 (2.9%)
Mean follow-up time (months), (median [P25, P75])	
<2 cm	48.94 [14.39, 73.54]
≥2 cm	34.79 [23.69,41.89]

This study mainly focused on the relation between the factors listed in Table 4 and the local recurrence rate. From Table 3 , considering the number of cases and the outcome of recurrence, surgical margins could be grouped into surgical margins <2 cm and ≥2 cm. The outcomes of this group led to significant differences in the recurrence rate, with 20.8% in <2 cm margin and 0.0% in ≥2 cm ( *p* = 0 *.157* ). The depth margin median was 1.20 cm, which had no statistical significance, as well as the pathological margin. However, the tumor size statistics significantly affected the local recurrence rate, with the mark at 9 cm ( *p* < 0.05). Other managements such as lymph node dissection, preoperative radiotherapy, postoperative radiotherapy, and postoperative chemotherapy had no statistical significance to local recurrence rate with the *p* -value of 1.000, 0.143, 1.000, and 1.000, respectively.

**Table 4:** The relation between local recurrence rate and other factors that might affect the recurrence, including surgical margin, deep margin, pathological margin, tumor size and area, LN dissection, other pre- and postoperative treatment

Factors	Local recurrence	No recurrence	*p* -Value
Surgical margin			0.157
<2 cm	5 (20.8%)	19 (79.2%)	
≥2 cm	0 (0.0%)	11 (100.0%)	
Deep margin (median [P25, P75])	1.20 [0.95, 1.95]	1.00 [0.80, 1.50]	0.410
Pathological margin			0.561
Negative	5 (17.2%)	24 (82.8%)	
Positive	0 (0.0%)	6 (100.0%)	
Tumor size			**0.026**
<9 cm	1 (4.2%)	23 (95.8%)	
≥9 cm	4 (36.4%)	7 (63.6%)	
Tumor size (median [P25, P75])	12.50 [6.60, 17.60]	7.00 [4.47, 8.85]	0.056
Tumor area (median [P25, P75])	111.25 [48.90, 224.75]	30.00 [14.50, 58.80]	**0.025**
LN dissection			1.000
No	4 (13.8%)	25 (86.2%)	
Yes	1 (16.7%)	5 (83.3%)	
Preoperative radiotherapy			0.143
No	4 (11.8%)	30 (88.2%)	
Yes	1 (100.0%)	0 (0.0%)	
Postoperative radiotherapy			1.000
No	4 (13.8%)	25 (86.2%)	
Yes	1 (20.0%)	4 (80.0%)	
Postoperative chemotherapy			1.000
No	5 (15.2%)	28 (84.8%)	
Yes	0 (0.0%)	1 (100.0%)	

**Fig. 1:**
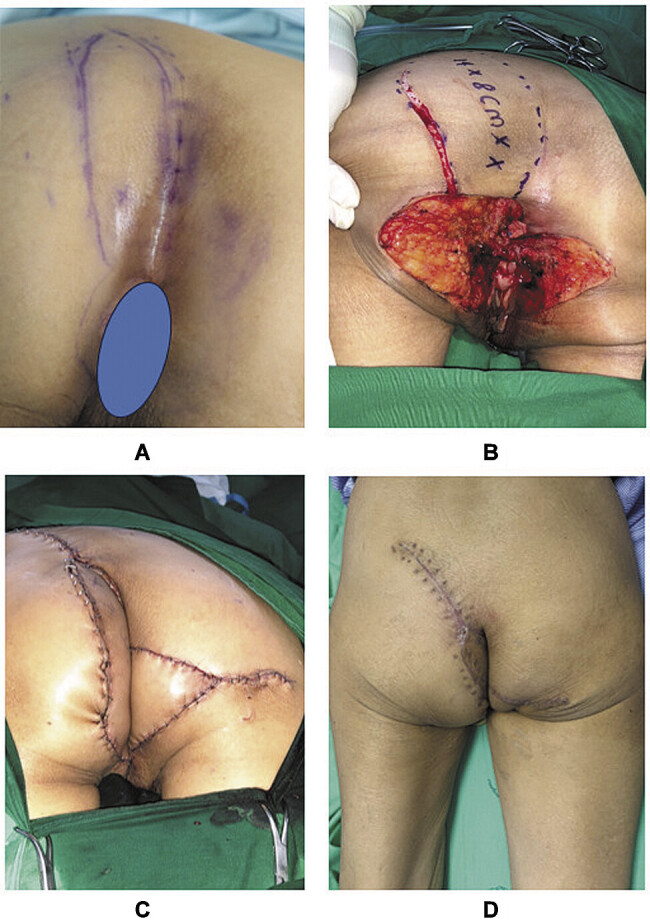
Relation of tumor-free rate and time to recurrence in years. The red line represents surgical margin ≥2 cm group. The blue line represents surgical margin <2 cm group throughout the 9-year period. The *p* -value of the log-rank test is *p* = 0.165.

**Fig. 2:**
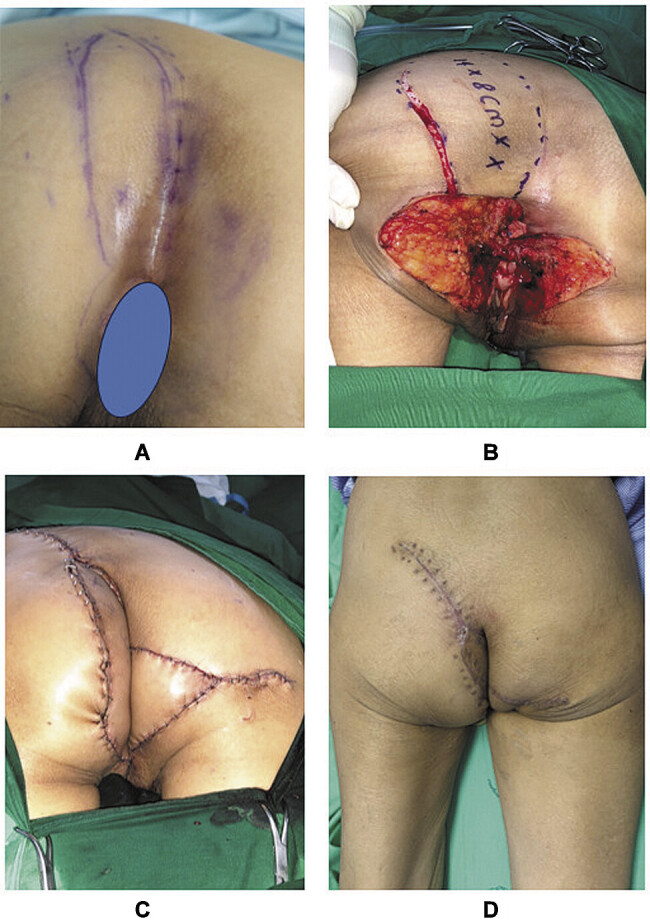
Patient pictures of vulva and perianal Paget's disease with a 4 × 7 cm lesion located at the left vulva and perianal area ( **A** ). Collaborative efforts among oncologists, and surgeons led to the decision for wide surgical excision with a 1-cm margin for adequate of excision and meticulous depth clearance of approximately 0.3 cm deep to the subcutaneous layer ( **B** ). The pathological tissue was reported with free margin from pathologist. Therefore, further surgery was not required. Reconstruction using a propeller flap from the left buttock ensured both functional and aesthetic restoration ( **C** ). Follow-up assessments at 1 month postoperatively ( **D** ) and at 42 months revealed all margins to be free of microscopic disease, reassuring regarding the adequacy of excision, while postoperative recovery was uneventful, highlighting successful wound healing and functional restoration.

The relation between the tumor-free rate and time to tumor recurrence is demonstrated in Fig. 1 below. The surgical margin ≥2 cm group has no recurrence in 9 years. The surgical margin <2 cm group showed recurrences throughout the follow-up period ( *p* = 0.165). Figure 2 shows the time series of the patient diagnosed with EMPD with a satisfying outcome until the follow-up of 42 months.

## Discussion

The results of this study showed that the surgical margin of 2 cm signified the outcome of local recurrence, with 20.8% in the <2 cm margin group and no local recurrence in the ≥2 cm margin group. Although the result showed a remarkable outcome, the statistic had no significance due to the small number of recurrence cases, with only 35 patients and 5 recurrence cases. The tumor size, in contrast, had statistically affected the rate of tumor recurrences ( *p* < 0.05). Other factors, such as deep surgical margin, pathological margin, lymph node dissection, and oncological treatments, showed no significant outcome correlated to the local recurrence rate.

A wider surgical margin indicated better surgical outcome; however, a wider margin also led to more tissue defects. 7 Hendi et al recommended a 5-cm surgical margin. 10 However, EMPD usually has a non-aggressive prognosis 14 ; excision up to 5 cm would make a large tissue defect in the patient, especially in the genitalia area. 15 Nevertheless, some factors which are mentioned in other studies may not have significance toward local recurrence rate in this study, for instance, deep dermal invasion, 9 radiation therapy, 16 age, 17 or surgical technique. 18 This controversial outcome might be from the limitation of sample size, which affected the statistical power of the study. Since the EMPD is a rare cutaneous lesion with low prevalence of cases, a larger-scale study was limited due to its incidence. Additional studies discussed the relation between the positive pathological margins and ill-defined borders to the recurrence rates, since EMPD usually has an ambiguous border. 19 20 In this study, whether the border is ill-defined or well-defined, free borders are all confirmed by the pathological margin; therefore, the positive pathological margin is of concern in this study. Many studies supported that positive pathological margin has higher recurrence rates, 19 21 while some showed that it is not statistically significant. 22 23 Correlated to Kato et al, where residual Paget's were found up to 47% with only a 5.9% recurrence rate (one case), our study also showed no relation between pathological margin and recurrence rate. As for tumor size, Wong et al also suggested a margin <2 cm for tumors less than 6 cm. 24 Although our study might not be statistically significant, the recurrence rates are still similar to the lowest rate in the study. Also, the study did not clarify the <2 cm margin, and which margin should be performed, thus combining with our study that a 1-cm margin had up to a 20.8% recurrence rate. Further study focusing on the margin of 1 to 2 cm should investigate deeply with a larger number of study cases, multicenter recruitment, and more recurrent cases should be performed for a better understanding of the surgical margin.

In conclusion, this retrospective study's objective is to indicate the optimal surgical margin for EMPD. The relationship between surgical margin and the recurrence rate of the disease was analyzed, showing that a margin of ≥2 cm gave a better outcome. Additionally, this study also indicated that a tumor of ≥9 cm would lean toward no local recurrence rate. From the study, it can be observed that tumor size could affect the recurrence rate, but with a surgical margin of more than 2 cm, there was no recurrence rate at all. Even without statistical proof, it could be implied that a surgical margin size of more than 2 cm was suggested for tumor-wide excision in EMPD patients.

### Conclusion

EMPD is a rare neoplastic cutaneous lesion that led to an inconclusive suggestion in the surgical margin. This retrospective analysis, conducted at Siriraj Hospital between 2008 and 2018, demonstrated the importance of the factors, including surgical margin and tumor size, in determining disease recurrence rate in Thai people. The study compared tumor size and disease outcome, showing that tumor size statistically affected the recurrence rates. Although larger tumor size correlated with the recurrence rates, resection with a surgical margin of 2 cm still yielded the outcome of no recurrence. Therefore, this study suggested a surgical margin of 2 cm as an optimal margin for excising the tumor.

In conclusion, this study's results supported the use of the surgical margin of ≥2 cm. This margin gave an outcome with no local recurrence throughout the 10-year period and also gave minimal tissue dissection. Although this study demonstrated a promising outcome, further studies should be conducted to obtain a proper number of EMPD cases for better statistical significance due to its low occurrences. Therefore, we suggested excising the EMPD tumor with a 2-cm margin for better outcomes and better patient quality of life.
